# A comprehensive genome-wide scan detects genomic regions related to local adaptation and climate resilience in Mediterranean domestic sheep

**DOI:** 10.1186/s12711-021-00682-7

**Published:** 2021-12-02

**Authors:** Valentina Tsartsianidou, Enrique Sánchez-Molano, Vanessa Varvara Kapsona, Zoitsa Basdagianni, Dimitrios Chatziplis, Georgios Arsenos, Alexandros Triantafyllidis, Georgios Banos

**Affiliations:** 1 Department of Genetics, Development & Molecular Biology, School of Biology, Aristotle University of Thessaloniki, 54124 Thessaloniki, Greece; 2 Division of Genetics and Genomics, School of Veterinary Studies, The Roslin Institute and Royal (Dick), University of Edinburgh, Easter Bush, Midlothian, EH25 9RG UK; 3 Department of Animal and Veterinary Sciences, Scotland’s Rural College, Roslin Institute Building, Easter Bush, Midlothian, EH25 9RG UK; 4 Department of Animal Production, School of Agriculture, Aristotle University of Thessaloniki, 54124 Thessaloniki, Greece; 5 Laboratory of Agrobiotechnology and Inspection of Agricultural Products, Department of Agriculture, International Hellenic University, Alexander Campus, 57400 Sindos, Greece; 6 Laboratory of Animal Husbandry, School of Veterinary Medicine, Aristotle University of Thessaloniki, 54124 Thessaloniki, Greece

## Abstract

**Background:**

The management of farm animal genetic resources and the adaptation of animals to climate change will probably have major effects on the long-term sustainability of the livestock sector. Genomic data harbour useful relevant information that needs to be harnessed for effectively managing genetic resources. In this paper, we report the genome characterization of the highly productive Mediterranean Chios dairy sheep and focus on genetic diversity measures related with local adaptation and selection and the genetic architecture of animal resilience to weather fluctuations as a novel adaptative trait linked to climate change.

**Results:**

We detected runs of homozygosity (ROH) and heterozygosity (ROHet) that revealed multiple highly homozygous and heterozygous hotspots across the Chios sheep genome. A particularly highly homozygous region was identified on chromosome 13 as a candidate of directional genetic selection associated with milk traits, which includes annotated genes that were previously shown to be linked to local adaptation to harsh environmental conditions. Favourable heterozygosity related with a potentially protective role against livestock diseases and enhanced overall fitness was revealed in heterozygous-rich regions on sheep chromosomes 3, 10, 13 and 19. Furthermore, genomic analyses were conducted on sheep resilience phenotypes that display changes in milk production in response to weather variation. Sheep resilience to heat stress was a significantly heritable trait (h^2^ = 0.26) and genetically antagonistic to milk production. Genome-wide association and regional heritability mapping analyses revealed novel genomic markers and regions on chromosome 5 that were significantly associated with sheep resilience to climate change. Subsequently, an annotation analysis detected a set of genes on chromosome 5 that were associated with olfactory receptor complexes that could participate in heat stress mitigation through changes in respiration rate and respiratory evaporation. Other genes were grouped in previously reported biological processes relevant to livestock heat dissipation, including stress and immune response.

**Conclusions:**

Our results may contribute to the optimal management of sheep genetic resources and inform modern selective breeding programmes that aim at mitigating future environmental challenges towards sustainable farming, while better balancing animal adaptation and productivity. Our results are directly relevant to the studied breed and the respective environmental conditions; however, the methodology may be extended to other livestock species of interest.

**Supplementary Information:**

The online version contains supplementary material available at 10.1186/s12711-021-00682-7.

## Background

Intensive genetic selection of farm animals combined with environmental changes raises concerns about the preservation of livestock genetic diversity. Modern programmes of selective breeding must integrate the effective management of genetic resources and the adaptation of animals to present and future challenges [1]. The integration of methods to mitigate genetic erosion and comprehensively evaluate animal performance through the characterization of adaptive traits that are relevant to climate change adaptation [2] would facilitate the sustainable management of livestock biodiversity [3].

Assessment of genome diversity, by estimating homozygosity and heterozygosity levels and genomic inbreeding, is of paramount importance for the management and conservation of genetic resources [4]. The availability of medium- and high-density genome-wide DNA arrays for commercial livestock species [5, 6] enables the estimation of multiple genomic diversity indicators. In particular, the effective quantification of individual homozygosity levels based on the detection of runs of homozygosity (ROH) [7, 8] could be used to assess and manage diversity and prevent inbreeding depression [9]. Estimation of inbreeding coefficients based on ROH is considered as a powerful approach that is widely used in biodiversity studies [10–12], and offers the possibility to distinguish between ancient and recent inbreeding [13]. In addition, the detection of ROH hotspots (also referred to as ROH islands) can identify suggestive genomic selected regions that harbour genes associated with animal domestication [14], breed formation [15], traits of economic importance [16], and local adaptation to new environments [12]. Furthermore, heterozygosity levels are commonly estimated as a genetic diversity indicator. Recently, it has been proposed that highly heterozygous regions throughout the genome, known as heterozygosity-rich regions (ROHet) or ROHet islands [17], are strongly associated with animal fitness and survival, and heterotic balancing selection processes [17–19].

The study of the genetic architecture of adaptive traits that are relevant to animal performance in response to changing environmental conditions, such as weather fluctuations, could also contribute to the characterization of animal genetic resources [20], and at the same time, provide essential information for future selective breeding strategies to mitigate the effects of climate change on the respective breeding goals [21, 22]. Genome-wide association studies on heritable adaptive traits have recently focused on animal production resilience to climate change in dairy cattle [23] and dairy goats [24], and have revealed potential candidate genes that are involved in animal stress and metabolic responses. Simulation studies have demonstrated that the integration of resilience traits in selective breeding programmes could enhance long-term sustainability of animal performance [22].

The present study focuses on the genomic characterization of the Chios dairy sheep, which is one of the most productive sheep breeds worldwide. Our specific objectives were to: (i) derive and assess multiple genetic diversity indicators including levels of homozygosity and heterozygosity, and genomic inbreeding estimates, (ii) detect genomic regions associated with local adaptation and selection based on ROH and ROHet hotspots, and (iii) characterize an adaptive trait relevant to climate change defined as the performance resilience of animals to weather volatility.

## Methods

### DNA extraction and genotyping

Total genomic DNA was extracted from blood samples of 600 Chios female sheep (milking ewes) from three farms in Northern Greece, according to the standard protocol of the QIAamp DNA Mini and Blood Mini kit (QIAGEN, USA), following the manufacturer’s recommendations with minor modifications. DNA samples were quantified using a Nanodrop spectrophotometer and stored at − 20 °C until use. Subsequently, DNA samples were oven-dried and 500 ng per sample were transferred to 96-well plates. All DNA samples were genotyped with the OvineSNP50 Genotyping BeadChip that contains 54,124 single nucleotide polymorphisms (SNPs) (Illumina, Inc., U.S.) and was developed in collaboration with the International Sheep Genomics Consortium [25] which included 23 Chios sheep.

Quality control of genotypes was performed by setting sample and marker call rate thresholds at 90% using the PLINK 1.07 software [26]. In addition, all SNPs on the sex chromosomes were removed resulting in a final dataset of 538 individuals and 51,124 SNPs spread across the 26 ovine autosomes. SNP positions were assigned according to the Oar_v4.0 sheep genome assembly [27].

### Genome-wide characterization

First, genotypes were analysed to derive observed (H_o_) and expected (H_e_) heterozygosity levels, and the inbreeding coefficient (*F*) based on the difference between the observed and expected number of homozygous genotypes under Hardy–Weinberg equilibrium, which were calculated from the allele frequencies in the sample and the total number of markers. These analyses were conducted with the PLINK 1.07 software [26].

Subsequently, ROH and ROHet were detected by implementing the R package detectRUNS [28] and applying the sliding-window approach for ROH and the consecutive method for ROHet detection [29]. The following input parameters were set to detect ROH based on a previous study for ROH identification that used medium-density SNP data [30]: (i) a sliding window size of 50 SNPs across the genome; (ii) a proportion of homozygous overlapping windows of 0.05; (iii) for each ROH, a minimum number of 20 consecutive SNPs and a minimum length of 250 kb; (iv) a maximum gap of 250 kb between consecutive homozygous SNPs; and (v) a maximum number of one missing call and one heterozygous genotype allowed in a ROH. Subsequently, highly homozygous regions, defined as ROH islands, were identified by extracting the 99.9 quantile of the frequency distribution of SNPs within a ROH.

The genomic inbreeding coefficient based on ROH (${F}_{ROH}$) was also estimated for each individual, as described by McQuillan et al. [31]:

$${F}_{ROH}=\frac{\sum {L}_{ROH}}{{L}_{aut}},$$

where $\sum {L}_{ROH}$ is the total length of all ROH in each individual genome and ${L}_{aut}$ is the total length of the autosomal genome according to the DNA array used for genotyping.

Contrary to ROH, to date the optimal parameters for ROHet detection have not been extensively studied. In the current study, we set the following input parameters: (i) for each ROHet, a minimum number of 15 consecutive SNPs and a minimum length of 250 kb; (ii) a maximum gap of 250 kb between consecutive homozygous SNPs; and (iii) a maximum number of two missing calls and three homozygous genotypes. Highly heterozygous genomic regions, defined as ROHet islands, were identified based on the frequency distribution of SNPs within a ROHet, as described for ROH islands above.

### Characterization of climate resilience

For the subsequent analyses, an additional edit was applied to the genotyping data, where SNPs with a minor allele frequency lower that 0.01 were removed. Thus, the remaining analyses were based on the same 538 individuals and the remaining 47,605 SNPs across the 26 ovine autosomes.

#### Genotype principal component analysis

A principal component analysis was performed on the animal genotypes to explore the potential population structure that should be accounted for in the following downstream genomic analyses to avoid possible inflation effects. The GEMMA software [32] was used for this purpose and the decomposed relatedness matrix between individuals was visualized.

#### Animal phenotypes

Milk yield records were available for all genotyped animals during the 2003–2018 period, during which animals had multiple lambings (births of lambs) followed by lactation periods (milking periods) lasting on average five months. Milk yield was recorded on each individual on a specific date once per month within a lactation. Thus, multiple milk yield records were available for each genotyped animal. This dataset comprised 10,348 records on 538 individuals.

Corresponding weather data were obtained from the Hellenic National Meteorological Service pertaining to the weather stations that were closest to the three farms on which animals were sampled. Average daily air temperature data were extracted for the dates corresponding to the available milk yield records.

Individual resilience phenotypes, reflecting a change in milk production in response to temperature variability, were derived based on a methodology that is described in detail in [33]. Briefly, reaction norm functions were fitted to milk yield records using a random regression model that included the corresponding air temperature measurement and accounted for the fixed effects of farm, lactation number, calendar year and month of lambing, and the number of days from lambing when milk yield was recorded. Resilience phenotypes were essentially the resulting slopes of individual reaction norm curves at different temperature levels. For the purposes of the present study, we focused on temperatures of 10 °C and 25 °C, which represent thresholds of cold and heat stress, respectively, for sheep raised in this geographic region. Animal resilience reflected the changes in milk yield in response to changes in temperature under cold and heat stress conditions. The above analyses were conducted using the BLUPF90 software [34] in RStudio with R.3.6.1 [35].

The newly derived resilience phenotypes were studied together with animal lifetime milk production, estimated from daily milk yield records according to previously described formula [36], and length of productive life, defined as the total number of days a ewe was milked.

#### Estimation of genomic parameters and breeding values

Variance components of animal resilience to temperature variability in cold and hot weather conditions were estimated to implement the following mixed linear model:

1 $$\mathbf{y}=\mathbf{W}{\varvec{\upalpha}}+\mathbf{Z}\mathbf{u}+{\varvec{\upepsilon}},$$

where $\mathbf{y}$ is the vector of animal phenotypes, ${\varvec{\upalpha}}$ is the vector of associated fixed effects, $\mathbf{u}$ is the vector of random polygenic (additive genetic) effects, which is distributed as a multivariate normal distribution $MVN({\bf{0}},{V}_{g}\mathbf{G})$ with $\mathbf{G}$ being the genomic relationship matrix and ${V}_{g}$ the genomic variance of the trait, $\mathbf{W}$ and $\mathbf{Z}$ are the corresponding design matrices, and ${\varvec{\upepsilon}}$ is the vector of random residual effect. Fixed effects included in Model (1) were: the farm on which the animals were raised, calendar year and month of first lambing, total number of lactations, and length of productive life; the first three principal components extracted from the population structure analysis described above were also included as covariates.

In separate analyses, variance components were also estimated for lifetime milk production and length of productive life using Model (1), with the addition of the latter as a covariate in the analysis of the former, and vice versa. Thus, results pertained to lifetime milk production adjusted for length of productive life, which reveals the true producing capacity of the animals. Furthermore, length of productive life was adjusted for the amount of milk produced, which then represents a functional longevity proxy. This trait reflects the capacity of an animal to remain on the farm independently of its production level, implying good health and fitness.

Genomic relationships between animals were estimated based on their SNP genotypes using the Regional Heritability Advanced Complex Trait Analysis (REACTA) software [37] and were incorporated in the analyses above. In each case, trait heritability was derived as the ratio of the estimated additive genetic variance to the total phenotypic variance.

Phenotypic and genomic correlations between the studied traits were subsequently estimated in a series of bivariate analyses with the above-described models. Statistical significance of all estimates was assessed using the two-tailed Student’s t-distribution.

The same model was also used to derive the genomic estimated breeding values (GEBV) of each individual for the studied traits. For resilience traits, these values represent the inherent propensity of animals to maintain milk production levels regardless of changing weather conditions. The respective GEBV accuracy was estimated as:

$$A\mathrm{c}curacy=\sqrt{1-\frac{PEV}{{\sigma }_{G}^{2}}},$$

where $PEV$ is the prediction error variance of the GEBV and ${\sigma }_{G}^{2}$ is the trait additive genetic variance. In this context, accuracy of the GEBV reflects the correlation of the GEBV with the true unknown genetic value of an animal. All these analyses were conducted using the ASReml 4.1 software [38].

#### Genome-wide association study

Animal phenotypes and genotypes were jointly analysed to identify SNPs associated with animal resilience to hot and cold weather, lifetime milk yield, and length of productive life. The same effects as in the above-described Model (1) were fitted, with the addition of the term $\mathbf{x}{\varvec{\upbeta}}$, with $\mathbf{x}$ being the vector of SNP genotypes and ${\varvec{\upbeta}}$ their associated effects. Analyses were conducted using the GEMMA software [32]. A Bonferroni correction was applied for multiple testing to determine a genome-wide (P < 0.05) and suggestive (one false positive per genome scan) significance threshold, resulting in final threshold values of P < 1.05E−06 and P < 2.10E−05, respectively, corresponding to −log10 (P) of 5.98 and 4.68, respectively. In order to account for further cryptic population structure that may inflate the test statistics, the inflation factor λ was used for correction [39].

#### Regional heritability mapping

Regional heritability mapping (RHM) was implemented to identify potential genomic regions associated with animal resilience and the other traits by scanning windows across the whole genome. A sliding-window approach was used with genomic regions of 100 consecutive SNPs (several window sizes were tested to evaluate the optimal region size) and overlaps of 30 SNPs defined along each autosome. The REACTA software [37] was used with the following model:

2 $$\mathbf{y}=\mathbf{W}\mathbf{a}+{\mathbf{X}\mathbf{u}}_{(i)}+{\mathbf{Z}\mathbf{u}}_{(-i)}+{\varvec{\upepsilon}},$$

where $\mathbf{y}$ is the vector of individual animal phenotypes, ${{\mathbf{u}}}_{(i)}$ is the vector of the random effect of region $i$, distributed as $MVN[\bf{0},{\mathrm{V}}_{\mathrm{g}(\it {i}\mathrm)}{\mathbf{G}}_{(\it{i}\mathrm)}]$ (with ${\mathrm{V}}_{\mathrm{g}(i)}$ and ${\mathbf{G}}_{(i)}$ being the genomic variance and the genomic relationship matrix corresponding to the SNPs in region $i$, respectively) and ${\mathbf{u}}_{(-i)}$ is the random polygenic effect excluding region $i$, distributed as $MVN[\bf{0},{\mathrm{V}}_{{\mathrm{g}}({-}\it{i}\mathrm)}{{\mathbf{G}}}_{({-}\it{i}\mathrm)}]$ (with ${\mathrm{V}}_{\mathrm{g}(-i)}$ and ${\mathbf{G}}_{(-i)}$ being the genetic variance and the genomic relationship matrix corresponding to all SNPs other than those in region $i$, respectively); all other effects are as described for Model (1).

The likelihood ratio test (LRT) was used to assess the significance of the effect of each genomic region. In total, 681 regions were tested across the genome resulting in a genome-wide significance threshold (P < 0.05) after Bonferroni correction for multiple testing corresponding to P < 7.34E−05 with −log10 (P) of 4.13 and a suggestive significance threshold (one false positive per genome scan) defined at P < 1.47E−03 with −log10 (P) of 2.83.

#### Gene and functional annotation analysis

The extent of linkage disequilibrium (LD) was determined in regions where (i) ROH or ROHet islands were detected, and (ii) genome-wide and suggestive significant SNPs were identified in genomic and RHM analyses. Pairwise LD estimation (r^2^) and visualization were carried out using the PLINK v1.07 [26] and Haploview 4.2 [40] software. Then, gene annotation was performed using the Variant Effect Predictor software [41] within distances of ± 250 kb and 1 Mb according to pairwise LD results of ROH and ROHet, and genomic and RHM analyses, respectively. Functional annotation was performed for all the identified genes within the respective regions using the UniProt database [42]. Functional enrichment analyses were conducted using the gProfiler genomic tool [43] and the DAVID software [44] to detect candidate annotated genes involved in biological processes with relevance to animal resilience to climate change and the other biological functions.

## Results

### Genome-wide characterization

**Fig. 1:**
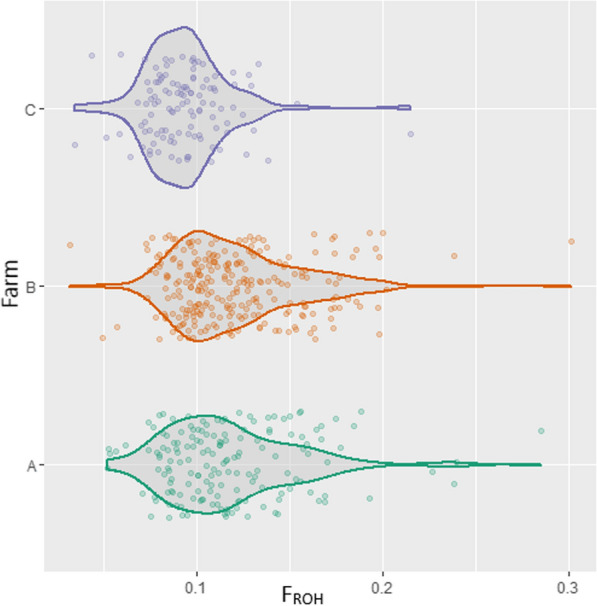
Individual run of homozygosity (ROH)-based inbreeding levels (*F*_*ROH*_) in Chios sheep. A, B, C correspond to the farm of origin of the animals

The average observed (H_o_) and expected (H_e_) heterozygosity estimates were 0.314 ± 0.167 and 0.355 ± 0.201, respectively, revealing quite high levels of polymorphism within Chios sheep breed. Average ROH-based inbreeding (${F}_{ROH}$) was 0.1133 and individual animal estimates ranged from 0.0314 to 0.3013. Estimates of the inbreeding coefficient (*F*) based on the difference between the observed and expected number of homozygous genotypes ranged from − 0.1123 to 0.2465, with an average of 0.005. Inbreeding levels were also estimated for different lengths of ROH segments and were lower for longer ROH (Table 1). Short ROH display ancient inbreeding and long ROH represent more recent inbreeding events [45]. High Pearson correlations were estimated between *F* and ${F}_{ROH}$ (0.97), and between coefficients of recent inbreeding (0.98), whereas lower estimates were obtained between recent and ancient inbreeding (0.78–0.80) (see Additional file 1: Fig. S1). Figure 1 illustrates the individual ROH-based inbreeding levels of Chios sheep according to farm of origin and shows that the population in farm C is less inbred, possibly implying a more intensive human-mediated directional selection on the other two farms.

**Table 1:** Average genomic (*F*) and ROH-based (*F*_*ROH*_) inbreeding coefficients

Inbreeding coefficient	Estimate (SE)
*F*	0.005 (0.002)
*F*_*ROH*_	0.113 (0.002)
*F*_*ROH 2–4 Mb*_	0.112 (0.002)
*F*_*ROH 4–8 Mb*_	0.075 (0.001)
*F*_*ROH 8–16 Mb*_	0.037 (0.001)
*F*_*ROH* >*16 Mb*_	0.020 (0.001)

*F* Based on observed and expected number of homozygous genotypes under the Hardy–Weinberg equilibrium, *F*_*ROH*_ estimates pertain to different size classes of the respective segment lengths

In total, 23,725 ROH were identified throughout the genome. The average number of ROH per animal was 44.1 and the average ROH length detected across all the autosomes was 4.92 Mb. The shortest ROH (736.2 kb) was detected on chromosome 14 and the longest (97.57 Mb) on chromosome 2. The highest individual sum of ROH reached 737.52 Mb with the corresponding maximum ${F}_{ROH}$ of 0.3013. The numbers of ROH identified by size class (length of ROH) are in Table 2.

**Table 2:** Number of identified ROH and ROHet per size class

	Size class (Mb)	Number
ROH regions	0–6	23,725
	6–12	5047
	12–24	1322
	24–48	179
	> 48	10

	Size class (kb)	Number
ROHet regions	250–500	469
	500–1000	13,820
	1000–2000	929
	0–6000	15,218

Numbers of runs of homozygosity (ROH) and heterozygosity (ROHet) pertain to different size classes of the respective segment lengths

Furthermore, 15,218 ROHet were identified in the studied sample of animals. The average ROHet length detected across all the autosomes was 719.92 kb; the shortest was detected on chromosome 1 (376.04 kb) and the longest on chromosome 10 (1748.15 kb). The numbers of ROHet by size class (length of ROHet) are in Table 2.

**Fig. 2:**
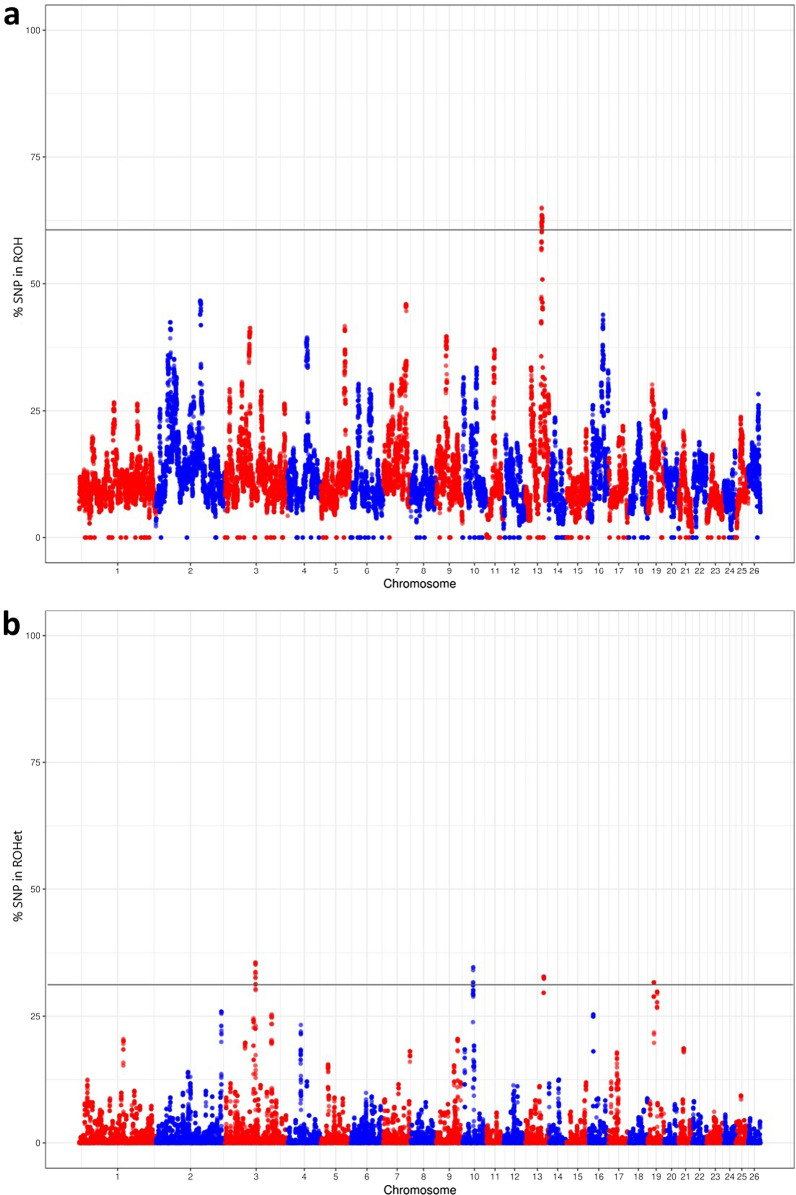
Manhattan plots showing the frequency of a SNP within homozygous and heterozygous segments in Chios sheep. **a** Runs of homozygosity (ROH), and **b** runs of heterozygosity (ROHet). Grey lines indicate the 99.9 quantile thresholds, defining the respective ROH/ROHet islands

Figure 2 illustrates the frequency of SNPs within a ROH or a ROHet across the autosomes, revealing homozygosity-rich and heterozygosity-rich genomic regions, respectively. These regions, termed ROH and ROHet islands, were identified by obtaining the 99.9 quantile of the frequency distributions of SNPs within a ROH or ROHet, corresponding to a frequency threshold of 0.61 and 0.32, respectively. Five such genomic regions were identified: (i) one ROH island representing a genomic region of 2.89 Mb on chromosome 13, where 55 SNPs and 57 genes are localized; and four ROHet islands, i.e. (ii) one (681.96 kb) on chromosome 3 including 15 SNPs, (iii) one on chromosome 10 (489.35 kb) including 12 SNPs, (iv) one on chromosome 13 (808.68 kb) including 16 SNPs and 25 annotated genes, and (v) one on chromosome 19 (479.36 kb) including nine SNPs and seven closely located genes. High LD levels were observed in all these chromosomal regions (see Additional file 2: Table S1).

### Functional annotation analyses—genomic characterization

Based on the average pairwise LD (r^2^) and average distance between the SNPs detected in the ROH and ROHet islands, a SNP annotation analysis was performed in 200–250 kb-regions upstream and downstream of the SNPs. Ninety-nine genes were located in the annotated regions of the respective ROH and ROHet islands (see Additional file 2: Table S1). Three of the candidate annotated genes located in the ROH island on chromosome 13 (*BMP7*, *ACSS2*, and *EPB41L1*) are known to be associated with animal traits, such as litter size in sheep [46] and milk quality in sheep and cattle [32, 47, 48], while the other genes (*CTCFL*, *PCK1*, *RAE1*, and *BMP7*) are reported to be involved in local adaptation processes [49]. Moreover, the annotated genes located in highly heterozygous regions (ROHet islands) on chromosomes 13 and 19, respectively, are potentially linked with animal immune response (*ROMO1*), protection against ruminant disorders (*LOC101117181*, and *SUMF1*), and cheese making traits (*ITPR1*) [50–53]. In addition, genes related to sheep domestication (*ITPR1* and *SUMF1*) [54] were found in the ROHet island on chromosome 19, implying positive selection on heterozygous genotypes. All the candidate genes that were identified in ROH and ROHet islands and that are associated with livestock performance and disease and with adaptation processes are summarized in Additional file 2: Table S1. All annotated genes and their molecular and functional characterization are also summarized in Additional file 3: Table S2.

## Characterization of climate resilience

### Population structure

**Fig. 3:**
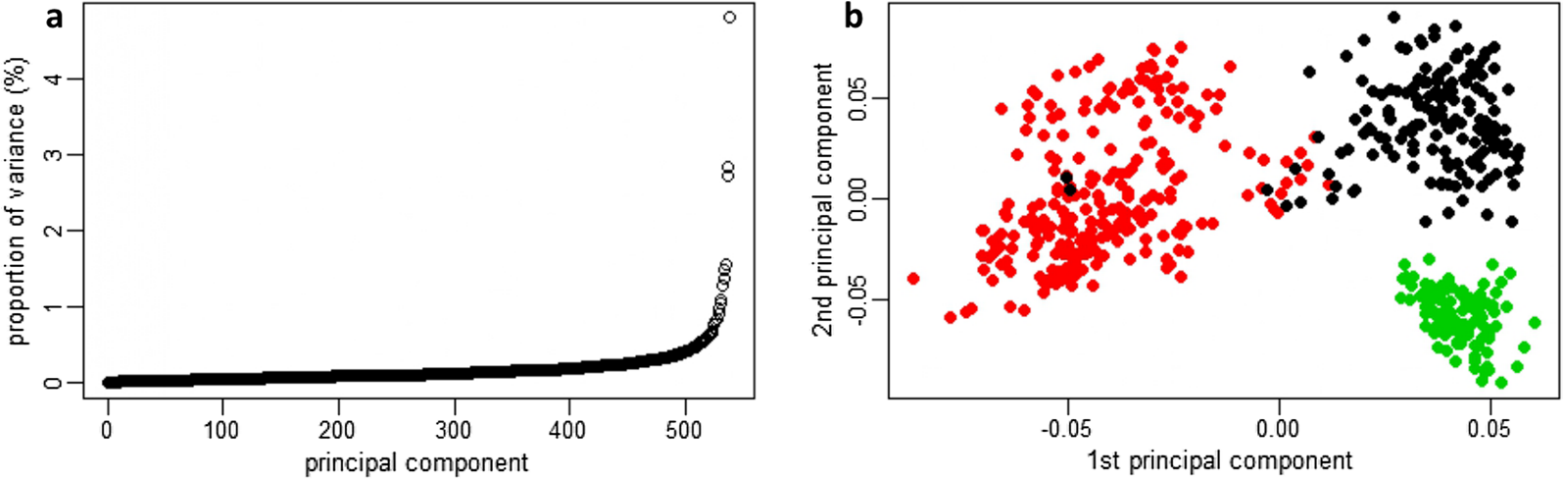
Principal component analysis (PCA) of Chios sheep. **a** Proportion of variation (%) corresponding to each principal component of the decomposed genomic relationship matrix; and **b** First and second principal components showing the population stratification attributed to the farm origin of individual sheep

The results of the principal component analysis that was carried out on the genomic relatedness matrix between individuals are illustrated in Fig. 3. The first three principal components accounted for almost 11% of the total variation (Fig. 3a). A strong population structure was revealed by the first two principal components and attributed to the farm of origin of animals (Fig. 3b). The latter may be explained by targeted genetic selection and mating practices on the different farms. A similar structure was revealed by the first and third principal components. Therefore, the first three principal components were fitted as covariates, together with the genomic relatedness matrix, in the following downstream genomic analyses to account for population structure.

### Animal resilience phenotypes

**Fig. 4:**
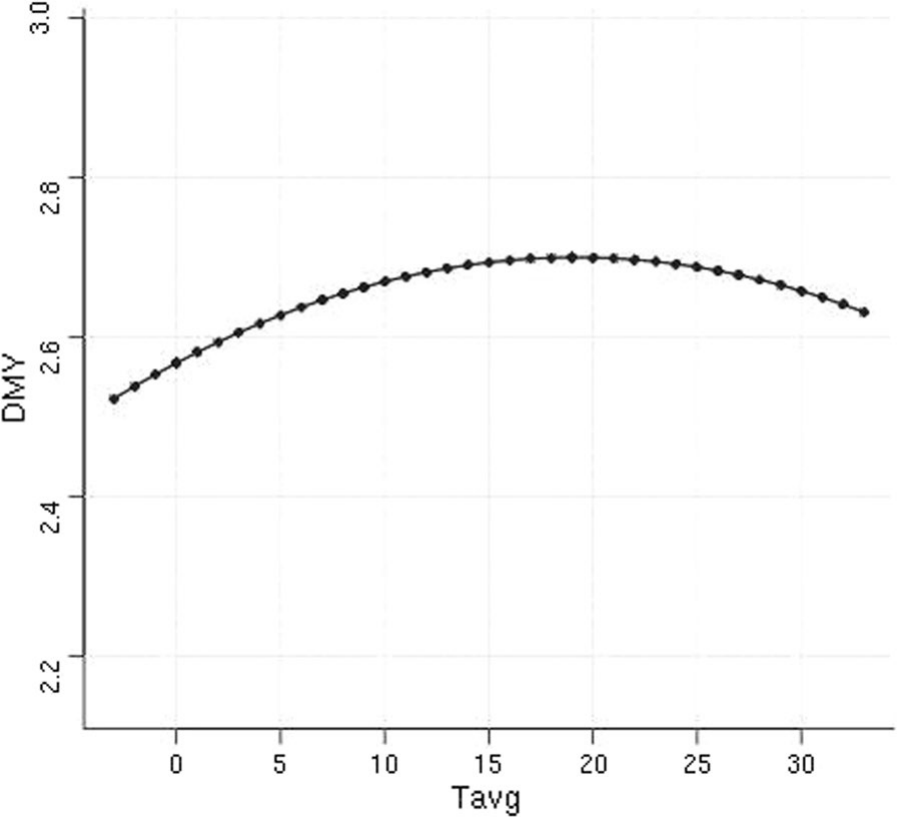
Chios sheep milk performance resilience to air temperature variation. The curve represents the average change in daily milk yield (DMY) in response to temperature variation (Tavg, °C)

Table 3 summarizes the statistics for the traits under study in the Chios dairy sheep dataset. The mean (SD) of average daily temperature was 15.51 °C (8.45). Figure 4 illustrates the average Chios sheep performance resilience to temperature fluctuations, which reflects the change in population daily milk yield in response to temperature volatility. Examples of individual animal performance resilience phenotypes represented by the slopes of reaction norm curves (see Additional file 4: Figure S2) reflect deviations from the population curve shown in Fig. 4.

**Table 3:** Descriptive statistics (mean and standard deviation) of the traits under study in the Chios dairy sheep dataset

Phenotype	Mean (SD)
Milk yield change by 1 °C temperature change at 10 °C (Resilience to cold weather)	0.003 (0.015)
Milk yield change by 1 °C temperature change at 25 °C (Resilience to hot weather)	− 0.006 (0.015)
Lifetime milk yield (kg)	1181.79 (768.35)
Length of productive life (days)	648.30 (373.85)

Indicative resilience phenotypes to cold (10 °C) and hot (25 °C) conditions were selected for further study. Positive or negative values of individual slopes represent milk yield changes in response to ambient temperature change, whereas zero (flat) slopes represent individuals whose milk production remains unaffected by temperature fluctuations.

### Genomic parameters of the traits under study

Trait heritability and correlation estimates are in Table 4. Heritability estimates of animal resilience to hot weather and lifetime milk yield were moderate and significantly higher than 0 (h^2^ = 0.20 and 0.26, SE = 0.095 and 0.009, respectively). Resilience to cold temperature and length of productive life were lowly heritable with estimates that did not reach statistical significance. The two animal resilience traits were strongly positively correlated at both the phenotypic and genetic levels. Phenotypic and genetic correlations of animal resilience under hot conditions with lifetime milk yield were negative, implying an antagonistic relationship between sheep production and fitness, while those between resilience to hot weather and length of productive life were positive as they both represent fitness traits. All other correlations were not statistically different from 0.

**Table 4:** Heritability (diagonal) and correlation (above: genomic, below: phenotypic) estimates between the traits under study with standard errors in parentheses

Phenotype	Tavg10	Tavg25	tmilk	tdim
Tavg10	0.03 (0.08)	0.87 (0.29)*	− 0.92 (0.67)	− 0.70 (0.95)
Tavg25	0.76 (0.02)*	0.20 (0.09)*	− 0.94 (0.07)*	0.76 (0.24)*
tmilk	− 0.39 (0.04)*	− 0.79 (0.02)*	0.26 (0.01)*	− 0.35 (0.80)
tdim	0.06 (0.05)	0.56 (0.04)*	0.07 (0.07)	0.05 (0.07)

*Tavg10* Tavg25: animal resilience at 10 °C and 25 °C, respectively, *tmilk* lifetime milk yield (kg), *tdim* length of productive life (days)

Estimates significantly different from 0 (P < 0.05) are indicated with an asterisk

### Genomic breeding values

**Fig. 5:**
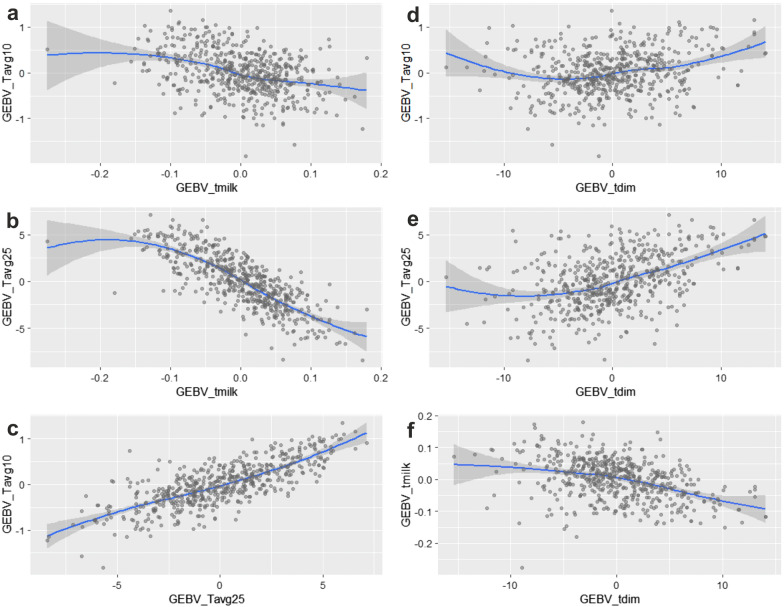
Comparison of estimated genomic breeding values (GEBV) of Chios sheep resilience to cold (Tavg10) and hot (Tavg25) weather conditions, lifetime milk production (tmilk), and length of productive life (tdim). **a** Resilience to cold and **b** hot weather compared to lifetime milk production respectively, **c** comparison between resilience to cold and hot weather conditions, **d** comparison of resilience to cold, **e** hot weather with length of productive life respectively, and **f** comparison between lifetime milk production and length of productive life

The comparison between individual GEBV estimated for the different studied traits is illustrated in Fig. 5. Notably, positive breeding values for resilience to cold and hot weather correspond to low genetic merit for lifetime milk yield and vice versa, implying a likely genetic antagonism for these traits, which is consistent with the negative genetic correlations in Table 4. The opposite is true for resilience to hot weather and length of productive life. In addition, high breeding values for length of productive life correspond to lower genetic merit for lifetime milk yield in accordance with the estimated negative correlation between these traits (Table 4).

Average GEBV accuracy estimates were 0.47, 0.20, 0.56 and 0.34 for animal resilience to hot and cold weather, lifetime milk yield, and length of productive life, respectively. The magnitude of these parameters reflects the corresponding trait heritability estimates summarized in Table 4. Higher heritability estimates are associated with more accurate GEBV.

### Genome-wide association study

**Fig. 6:**
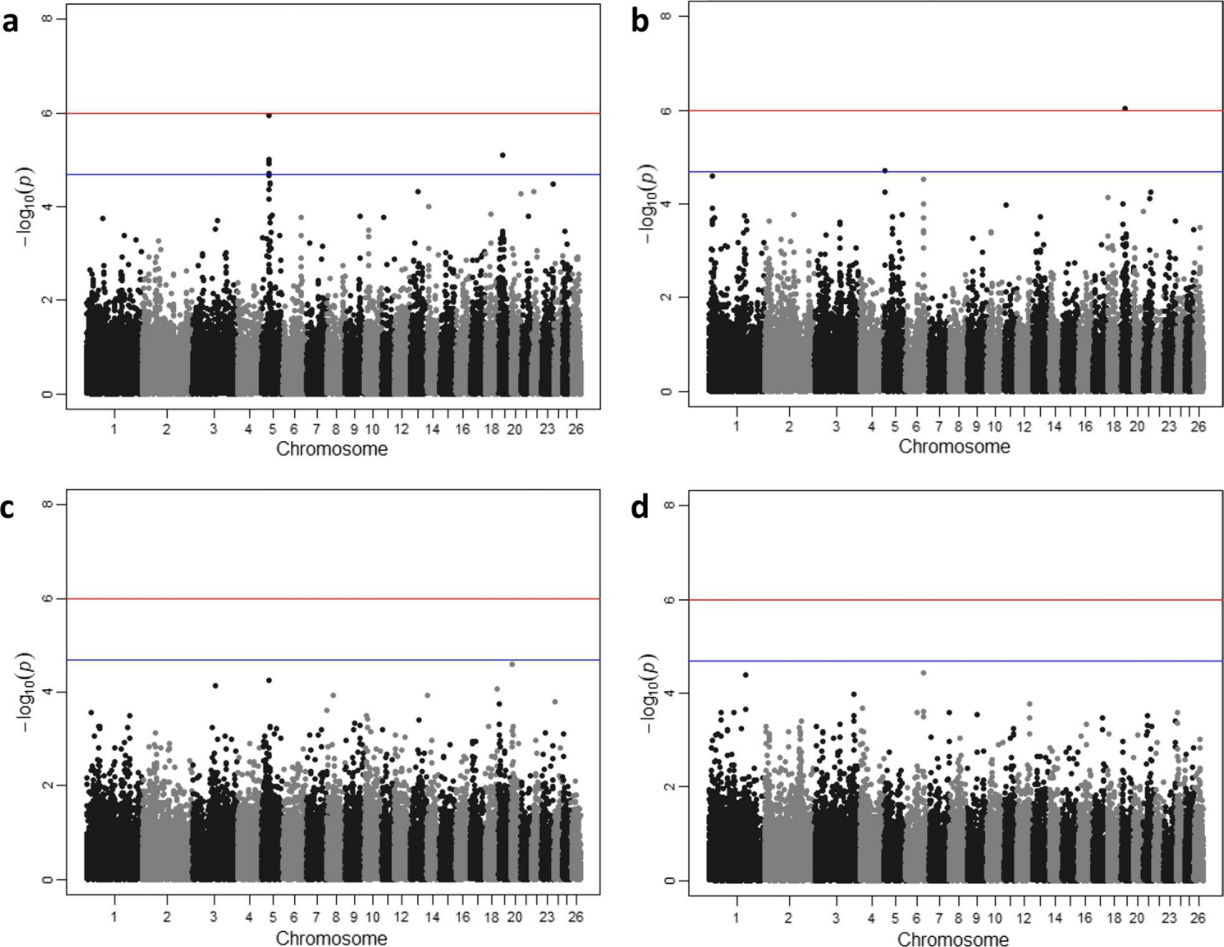
Genome-wide association results for Chios sheep resilience to temperature change and performance-related traits. **a** Resilience to hot weather conditions, **b** resilience to cold weather conditions, **c** lifetime milk yield, and **d** length of productive life. Red and blue lines indicate the genome-wide and suggestive (one false positive per genome scan) significance thresholds, respectively

Results from the GWAS of animal resilience under hot and cold conditions, lifetime milk yield, and length of productive life are illustrated in Fig. 6. The corresponding Quantile–Quantile (Q–Q) plots are provided in Additional file 5: Figure S3 and show no significant inflation. The results reveal a largely polygenic mode of inheritance for these traits, with specific genomic regions of interest located on chromosomes 5 and 19 for resilience to hot weather and lifetime milk performance.

One SNP approached the genome-wide significance threshold (P = 1.15E−06) and five genome-wide suggestive significant SNPs were also detected for resilience to hot weather. Interestingly, one of the latter on chromosome 19 also had a genome-wide significant association with lifetime milk yield with a substitution effect of opposite sign compared to resilience (Table 5), in concordance with the negative genetic correlation between the two traits (Table 4; Fig. 5). No significant SNP associations were detected for resilience to cold weather and length of productive life, consistently with the non-significant heritability estimated for these traits (Table 4).

**Table 5:** Genome-wide^a^ and suggestive significant SNPs associated with animal resilience to temperature change in hot weather and lifetime milk yield

Phenotype	Chr	SNP	Position (bp)	MAF	Beta coefficient (SE)	P-value
Resilience to hot weather	5	OAR5_38936884.1	35176238	0.32	− 0.005 (0.001)	1.15E−06
		OAR5_40288441.1	36496751	0.45	− 0.004 (0.001)	9.92E−06
		OAR5_41355579.1	37587760	0.34	− 0.004 (0.001)	1.17E−05
		s46299.1	36569778	0.32	− 0.004 (0.001)	1.20E−05
		OAR5_40166532_X.1	36373243	0.46	− 0.004 (0.001)	1.98E−05
	19	s14444.1	18983777	0.12	0.007 (0.002)	1.85E−05
Lifetime milk yield	19	s14444.1^a^	18983777	0.12	− 141.249 (28.432)	9.17E−07

*Chr* chromosome, *MAF* minor allele frequency, *Beta coefficient* substitution effect, *σ*^*2*^_*A*_*(SNP)* proportion of additive genetic variance attributed to detected SNPs

a: additive genetic effect and d: dominance effect (significantly different from zero (P < 0.05) are indicated with double asterisk); SE: standard error

The respective chromosomal locations refer to the Oar v4.0 genome assembly

### Regional heritability mapping

**Fig. 7:**
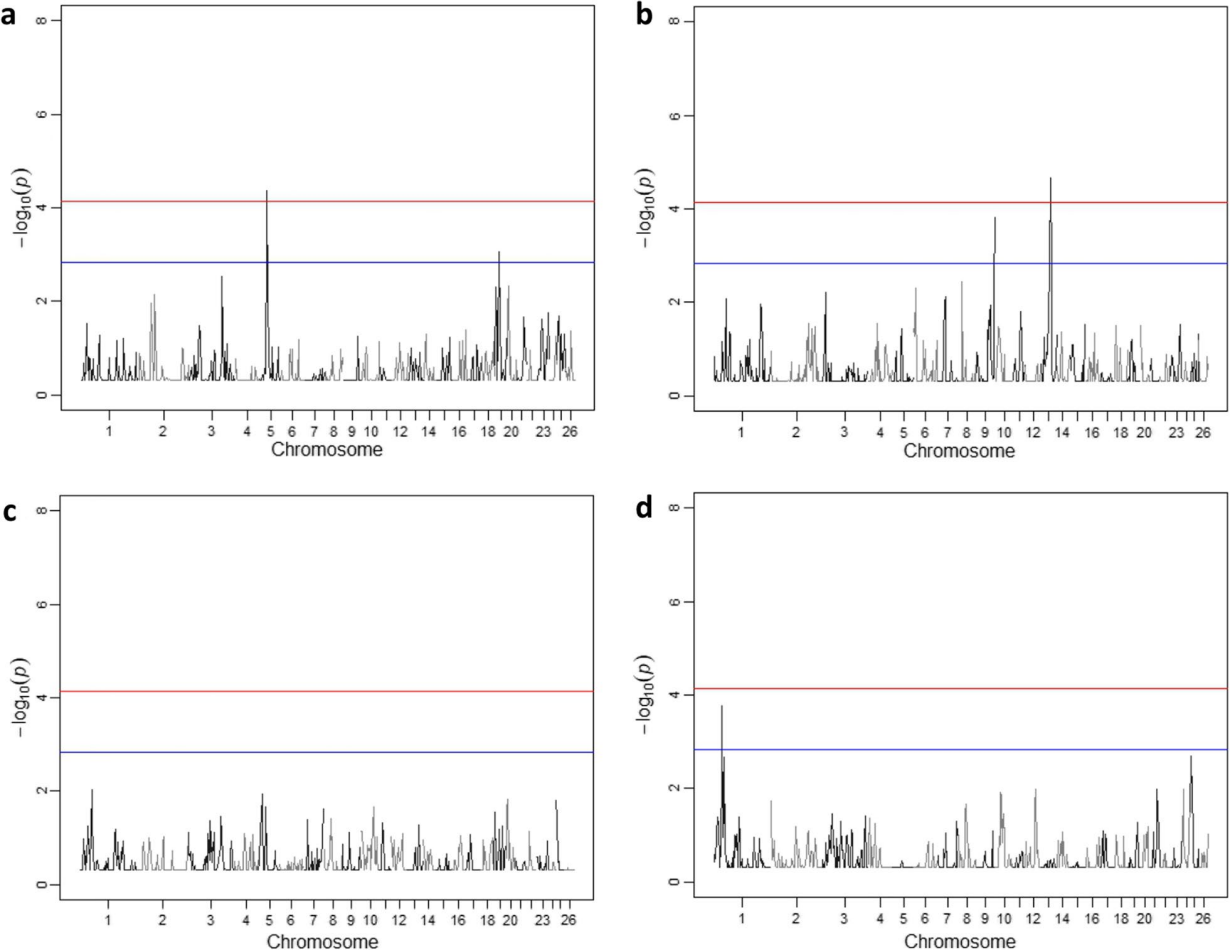
Regional heritability mapping results for Chios sheep resilience to temperature change and performance-related traits. **a** Resilience to hot weather conditions, **b** resilience to cold weather conditions, **c** lifetime milk yield, and **d** length of productive life. Red and blue lines indicate the genome-wide and suggestive (one false positive per genome scan) significance thresholds, respectively

Two neighbouring genomic regions were identified on chromosome 5 that exceeded the genome-wide and suggestive significance threshold (Fig. 7 and Table 6) for resilience to temperature change under hot weather conditions. Four of the five significant SNPs detected by GWAS were located within one region and the fifth was located in the other. Respective regional heritability estimates ranged from 0.06 to 0.07 (Table 6). Furthermore, a region exceeding the suggestive threshold was detected on chromosome 19, including SNP s14444.1, which was suggestive significant in the GWAS.

**Table 6:** Regional heritability (h^2^_reg_) for the detected genomic regions significantly associated with animal resilience to temperature change in hot weather, lifetime milk yield, and length of productive life

Phenotype	Chromosome	Genomic region—position (bp)		LRT	P-value	h^2^_reg_ (SE)
		Start	End			
Resilience to hot weather	5	37233570	43971641	11.570	3.35E−04	0.06 (0.03)
	5*	33896123	39299375	15.446	4.24E−05	0.07 (0.04)
	19	18588790	24437227	9.800	8.73E−04	0.05 (0.03)
Lifetime milk yield	13*	44169357	50958664	16.719	2.17E−05	0.05 (0.08)
Length of productive life	1	40313559	45665836	12.813	1.72E−04	0.06 (0.03)

*LRT* likelihood ratio test, *SE* standard error

Genome-wide significant regions are indicated with an asterisk

A genomic region located on chromosome 13 was detected for lifetime milk yield exceeding the genome-wide significance threshold, although this region did not include any SNPs identified as significant in the GWAS. A suggestive significant genomic region was detected on chromosome 1 for length of productive life. RHM analysis of animal resilience under cold temperatures did not reveal any genome-wide or suggestive significant associations.

### Functional annotation analyses—climate resilience

The LD structure of the region on chromosome 5 where the significant SNPs were identified in the genomic analysis of animal resilience to temperature change in hot weather was estimated (see Additional file 6: Figure S4). According to the results of pairwise LD, a SNP annotation analysis was performed within 1-Mb regions upstream and downstream of each SNP, and 94 candidate genes were detected in this region (see Additional file 7: Table S3). Then, this gene list was used as input to the functional enrichment analysis. The resulting protein interaction network includes all the identified candidate genes (see Additional file 8: Figure S5). Some of the identified biological processes are relevant to adaptation to ambient temperature fluctuations and to olfactory processes (see Additional file 9: Figure S6). Indicatively, the *olfactory receptor 56-like* and *Olfr56* genes are located 97,975 and 167.41 kb upstream of the suggestive significant SNP OAR5_41355579.1, whereas the *olfactory receptor 7A17-like* gene is located 212.78 kb downstream. In addition, several other annotation clusters were detected (see Additional file 10: Table S4) including genes that participate in biological processes that are related with stress response, such as the glucose, energy and protein metabolism (*B4GALT7*, *HK3*, *FAF2*, *MGAT1*, and *LMAN2*), and immune response (*BTNL9*, *TRIM41*, *TRIM52*, *FAF2*, and *PRR7*) [55, 56].

## Discussion

The present study was set out to provide a first insight into the genome of the highly productive Chios dairy sheep breed, including an assessment of its genetic diversity, identification of regions related with adaptation and selection, and dissection of the genomic architecture of an adaptive trait linked to animal resilience to climate change.

We derived multiple genome-wide indicators of the Chios genome diversity. Average observed and expected heterozygosity levels (H_o_ = 0.31, H_e_ = 0.35) were consistent with previously reported estimates for domestic sheep breeds worldwide [57] and particularly with other dairy sheep breeds raised in the Mediterranean region (H_e_ = 0.38–0.39) [12, 58]. By extension, this applies to expected and observed homozygosity levels which were used to derive the genomic inbreeding coefficient (*F*) shown in Table 1. Moreover, estimated average ROH-based inbreeding levels (${F}_{ROH}$) were moderate (Table 1 and Fig. 1) and within the range of previous ROH-based estimates in domestic dairy sheep (0.04–0.15) [12, 59, 60]. It should be noted here that these inbreeding coefficients collectively reflect genetic diversity levels in the population, but individually have different definitions and scales of measurement. Thus, *F*_*ROH*_ is an identity by descent probability ranging from 0 to 1, whereas, *F* is estimated based on allele frequencies resulting in both positive and negative values; this may explain the near zero mean value of the latter in our data (Table 1).

Inbreeding levels in farmed livestock are often attributed to selective pressure towards enhanced productivity resulting in the likely selection of and mating between genetically-related individuals. Long-term unmonitored selection towards the same direction, for example increased productivity, may result in increased homozygosity and compromise animal fitness [61]. In the present study, the strong population structure attributed to farm origin (Fig. 3b) implies mainly within-farm animal genetic selection and reproduction.

Patterns of homozygosity were observed according to different clusters of ROH by size class (Table 2 and Fig. 2a). Short to medium size ROH (0–6 Mb) constitute the majority of the identified homozygous segments (78%) and are indicative of ancient inbreeding events [31] or random recombination events in the respective genomic regions [62]. Long ROH were also present (Table 2), which indicate recent common ancestry due to a low probability of recombination events and selective pressure [14, 62].

We detected a genomic region with increased homozygosity, defined as a ROH island, spanning 2.89 Mb on chromosome 13 of the Chios sheep genome (Fig. 2a) (see Additional file 2: Table S1). This ROH island coincides with a genomic region previously identified as a signature of selection in European dairy sheep breeds [63], where quantitative trait loci (QTL) associated with milk quality traits are localized. These results suggest that a common selective pressure occurred independently in different domestic dairy sheep breeds.

Contrary to ROH islands, heterozygosity-rich regions (ROHet islands), which may be associated with balancing selection favouring animal fitness, are poorly characterized in livestock and, to our knowledge, have not been previously examined in Mediterranean sheep [64]. In this study, we observed patterns of clustered heterozygosity that reveal highly polymorphic regions of different lengths across the genome (Table 2 and Fig. 2b). Regions of 500 to 1000 kb constituted the majority of such heterozygosity-rich segments (91%). The maximum length of an identified ROHet island was 2 Mb, which is consistent with previously reported results for cattle [10] and horse [19] breeds. It is worth mentioning that the detection of ROHet and the respective selected parameters have not been extensively studied yet. Indeed, to date only one study including a sensitivity analysis on specific parameters, such as the numbers of missing and homozygous genotypes allowed within a ROHet segment, has been performed on cattle, suggesting that these regions could be characterized as heterozygosity-rich regions with expected homozygous loci distributed within the segments [10].

We detected four ROHet islands on ovine chromosomes 3, 10, 13 and 19, providing signals of favoured heterozygosity related to protection against livestock diseases, immune response, and local adaptation. Particularly, the heterozygous-rich segment on chromosome 10 (489.35 kb) is mapped to a selection sweep region that was previously identified in Egyptian sheep and includes a region of conserved synteny between Egyptian sheep and goat breeds adapted to hot arid environments [65]. Adaptation to such extreme local environments may involve the presence of multiple heterozygous genotypes associated with improved animal fitness in the respective conditions. Furthermore, the ROHet island located on chromosome 13 includes a region that was previously characterized as a signature of selection in local Russian sheep [66] and is related to mammary gland function and milk fatty acid composition [48]. The observed heterozygosity in this region may reflect a favourable impact on udder health compared to homozygous recessive genotypes.

Functional annotation analysis of the detected ROH island on chromosome 13 revealed a set of genes previously characterized as signatures of selection for local adaptation to harsh environmental conditions (*CTCFL*, PCK1, *RAE1*, *BMP7*, and *SPO11*) in sheep [49], and animal traits of economic interest related with production and mammary gland development and differentiation (*NFS1* and *TFAP2C*) [63, 67]. Interestingly, the *BMP7* and *TFAP2C* genes are also associated with lifetime milk production in goats [24]. This highly homozygous chromosomal segment may indicate a region of positive selection possibly attributed to selective breeding for increased milk production. Future comparative ROH studies on other dairy sheep breeds raised in similar environments could clarify the importance of this genomic region. Furthermore, annotated genes located within or close to the identified ROHet islands were mostly associated with immune response, protection against livestock disorders, and other animal performance traits (see Additional file 2: Table S1). The detected ROHet islands imply that heterozygosity is favoured for these genomic regions involved in common livestock diseases. For example, QTL related to susceptibility to ovine transmissible spongiform encephalopathy [68] and bovine spongiform encephalopathy [69] are located close to a ROHet island detected in the present study. This ROHet island also harbours genes that are associated with other disorders (*LOC101117181* and *CEP250*), metabolic pathways (*EIF6*), and milk-related traits (*ACSS2*, and *EPB41L1*), which suggest a multiple protective role. Highly heterozygous regions may also be associated with natural selection contributing to overall fitness in contrast with the ROH islands where directional genetic selection possibly leads to high levels of homozygosity.

To further investigate Chios sheep adaptiveness to changing environmental conditions, we evaluated the performance resilience of animals to weather fluctuations (Fig. 4) with a view to possibly include adaptive traits in future selective breeding schemes. For this purpose, we derived slopes of reaction norm functions fitted to random regression models, which reflect changes in individual milk production in response to changing daily temperatures (see Additional file 4: Fig. S2). The effect of heat stress is of particular concern to Mediterranean sheep production [56, 70, 71]. Notably, current and projected scenarios of climate change in this geographic area are suggestive of rising air temperature and increased weather volatility [72]. The heritability estimate of Chios sheep resilience to temperature change in heat stress conditions was significantly higher than zero (Table 4). Although these genomic estimates were based on a relatively small number of genotyped individuals, they are consistent with previous population estimates derived from a pedigree analysis of approximately 37,000 animals [33]. These results confirm that this trait could be improved through genetic selection. However, caution needs to be exercised when combining resilience with other animal traits in selective breeding. Phenotypic and genetic correlations between traits will determine the optimal way of combining them towards holistic animal improvement. In the present study, an antagonistic genetic correlation of resilience to hot weather with lifetime milk production was estimated (Table 4), which is consistent with previous estimates between animal resilience and milk production using pedigree data [33, 73]. This genetic antagonism was also evident in the estimated genomic breeding values for the two traits (Fig. 5), with sheep that are genetically predisposed for the highest milk production having negative values for resilience and length of productive life. This means that if selective breeding continues to focus on improving milk production, resilience to hot weather will decrease, as exemplified by reduced milk yield under heat stress conditions. Length of productive life that reflects animal longevity, would also decrease. Nevertheless, the results on length of productive life should be viewed with caution because of the lack of statistically detectable genetic variation for this trait in the studied population. These results imply the need for a multi-trait breeding index to underpin improvement in both animal production and fitness traits, where the latter are manifested in milk yield being unaffected by weather volatility and long lifespan. The feasibility of developing such indices in selective breeding given the appropriate input parameters has been documented [22].

Our genome-wide association (GWAS) and regional heritability mapping (RHM) analyses identified several SNPs, regions, genes, and molecular pathways associated with Chios sheep resilience under hot weather conditions (Figs. 6a and 7a, and Tables 5 and 6). These results may be used to enhance the accuracy of derived genomic breeding values in a selective breeding programme. In addition, one common SNP was associated with both resilience to hot weather and lifetime milk production and had an opposite substitution effect (Table 5), which is consistent with the above-mentioned genetic antagonism between the two traits. This information should be considered when combining these two traits in a breeding index for selection purposes. It is worth mentioning that very few genomic studies on animal resilience to climate change have been conducted [23, 24] and none of these reported regional heritability mapping results. Regional heritability mapping has been proposed as a complementary approach to detect genomic regions harbouring causative alleles of small effect that contribute to trait variation and could not be captured individually by GWAS [74]. Characteristically, our RHM results detected genomic regions on chromosomes 5 and 19 that are related to animal resilience to hot weather and included significant SNPs from the GWAS. In addition, RHM revealed significant peaks on chromosomes 1 and 13 that are associated with length of productive life and lifetime milk production, respectively, but where GWAS had not identified individual significant SNPs.

Our genomic analyses revealed regions accounting for a detectable variation in the studied traits (h^2^ = 0.05–0.07 for resilience under hot weather, h^2^ = 0.05 for lifetime milk production and h^2^ = 0.06 for length of productive life). Nevertheless, the majority of the trait variance was always accounted for by the polygenic effect. These results are consistent with previous studies, which suggest that fitness-related traits, such as resilience, are usually complex polygenic traits [24]. In the present study, the polygenic effect explained 15% of the variance of resilience to hot weather and 10 to 30% of the variance of the other traits.

Following the genomic analyses of animal resilience to climate change, we performed a functional enrichment analysis, which identified a set of 10 genes encoding olfactory receptors that are involved in multiple biological processes including detection of chemical stimuli in sensory perception and olfactory receptor activity and transduction (see Additional file 9: Fig. S6). Olfactory receptors are expressed in olfactory sensory neurons and detect volatile odorants in smells. However, some receptors are also expressed in other tissues [75, 76]. Response of small ruminants (sheep and goats) to elevated ambient temperatures is largely directed to respiratory evaporation through the respiratory tract in order to dissipate the increased heat load [71, 77, 78]. Heat stress has been related with inducible hypoxia [79]. The OAR5_38936884.1 SNP that we detected on chromosome 5 is located 157 kb upstream the annotated *HIG1* gene, which is a member of the hypoxia-inducible domain family and encodes a protein participating in the mitochondrial respiratory chain that catalyses the reduction of oxygen to water. A previous study [80] reported transcriptomic results on the activation of olfactory receptors during hypoxia, which leads to the stimulation of hyperventilation (increased respiration rate) and consequent respiratory evaporation and heat loss. Previously reported findings [81] proposed that Hsc70t, a variant of the heat shock protein 70 (Hsp70), which plays a key role in mitigating heat stress, enhances the expression of mammalian olfactory receptors. Therefore, we suggest that the above genes may constitute the hypoxia sensor in the breathing circuit that increases the respiration rate of the animals and results in thermal homeostasis through respiratory evaporation. Several other candidate genes were also grouped in annotation enrichment clusters (see Additional file 10: Table S4) that are associated with biological processes linked to animal endurance to heat stress [56]. Further research on the transcription of these genes may shed new light into the molecular mechanism that controls sheep resilience to heat stress.

## Conclusions

Our results offer a genome-wide understanding of sheep performance resilience to changing weather as a promising adaptive trait for future inclusion in selective breeding programmes. We have identified several genes that contribute to animal milk performance and overall fitness, and genomic regions that are linked to local adaptation of domestic sheep. We propose the integration of different genomic approaches to unravel the genomic regions that are important for breeding purposes as well as for the conservation of genetic variation within livestock breeds in terms of climate change mitigation and improvement of sustainability.

## Supplementary Information

**Additional file 1: Figure S1.** Correlation heatmap between different measures of inbreeding coefficient. The respective coefficients are: SNP-based genomic inbreeding (*F*), whole-genome ROH-based inbreeding (*F*_*ROH*_), and *F*_*ROH*_ by the respective size classes (ROH segment length) 2 to 4 Mb (FROH2_4), 4 to 8 Mb (FROH4_8), 8 to 16 Mb (FROH8_16), >16 Mb (FROH16).

**Additional file 2: Table S1.** Islands of runs of homozygosity (ROH) and heterozygosity (ROHet), and associated genes and animal traits of the studied sheep. The respective chromosomal locations are attributed to Oar v4.0 genome assembly.

**Additional file 3**: **Table S2.** Functional characterization of annotated genes in detected islands of runs of homozygosity (ROH) and heterozygosity (ROHet).

**Additional file 4: Figure S2.** Examples of individual animal resilience phenotypes manifested in reaction norm curves. Each curve represents the individual animal change in daily milk yield (milk, kg) in response to average air temperature variation on the day of milk record (Temperature, ^o^C).

**Additional file 5: Figure S3.** Quantile-Quantile plots for genome-wide association. Figures show the Q-Q plot before correction by the inflation factor for animal resilience to temperature change in hot weather (a), lifetime milk yield (b), length of productive life (c) and cold weather (d).

**Additional file 6: Figure S4.** Linkage disequilibrium (r^2^) heatmap of chromosome 5 region associated with Chios sheep resilience to hot weather conditions. This region extends between the furthest SNPs above the genome-wide and suggestive significance thresholds spanning a region of 2.41 Mb for animal resilience to temperature change in hot weather. The pairwise LD between these two SNPs is illustrated at the bottom right part of the figure.

**Additional file 7: Table S3.** List of the genes identified in the genomic analysis of resilience to temperature change under hot weather conditions. The respective genes were detected 1 Mb upstream and downstream of the genome-wide and suggestive significant SNPs.

**Additional file 8:** **Figure S5.** Protein interaction network. The present network represents the candidate identified genes for sheep resilience to temperature fluctuations under hot weather.

**Additional file 9:** **Figure S6.** g:GOSt multi-query Manhattan plot of functional enrichment analysis for sheep resilience to temperature fluctuations under hot weather. The X-axis represents the functional terms colour-separated by data source. The inset table summarizes the biological processes of the genes.

**Additional file 10:** **Table S4.** Significantly enriched annotation clusters and functional terms.

## Data Availability

Data analysed during the current study are available from the corresponding author on reasonable request. Phenotypic and genotypic datasets generated are deposited in Mendeley public repository available at https://data.mendeley.com/datasets/88s6zbf29w/1.
