# Supplementary material for: A comprehensive genome-wide scan detects genomic regions related to local adaptation and climate resilience in Mediterranean domestic sheep
Source: Genet Sel Evol. 2021 Dec 2;53:90. doi: 10.1186/s12711-021-00682-7 (PMC8641236; doi:10.1186/s12711-021-00682-7)
Supplement: Supplementary file 4 — Additional file 4: Figure S2. Examples of individual animal resilience phenotypes manifested in reaction norm curves. Each curve represents the individual animal change in daily milk yield (milk, kg) in response to average air temperature variation on the day of milk record (Temperature, oC). [file 12711_2021_682_MOESM4_ESM.docx]

**A comprehensive genome-wide scan detects genomic regions related to local adaptation and climate resilience in Mediterranean domestic sheep**

Valentina Tsartsianidou^1*^, Enrique Sánchez-Molano^2^, Vanessa Varvara Kapsona^3^, Zoitsa Basdagianni^4^, Dimitrios Chatziplis^5^, Georgios Arsenos^6^, Alexandros Triantafyllidis^1^ & Georgios Banos^3,6^

^1^ Department of Genetics, Development & Molecular Biology, School of Biology, Aristotle University of Thessaloniki, 54124 Thessaloniki, Greece

^2^ Division of Genetics and Genomics, The Roslin Institute and Royal (Dick) School of Veterinary Studies, University of Edinburgh, Easter Bush, Midlothian EH25 9RG, UK

^3^ Department of Animal and Veterinary Sciences, Scotland’s Rural College, Roslin Institute Building, Easter Bush, Midlothian EH25 9RG, UK

^4^ Department of Animal Production, School of Agriculture, Aristotle University of Thessaloniki, 54124 Thessaloniki, Greece

^5^ Laboratory of Agrobiotechnology and Inspection of Agricultural Products, Department of Agriculture, International Hellenic University, Alexander Campus, 57400 Sindos, Greece

^6^ Laboratory of Animal Husbandry, School of Veterinary Medicine, Aristotle University of Thessaloniki, 54124 Thessaloniki, Greece

**Corresponding author:** Valentina Tsartsianidou. ^1^ Department of Genetics, Development & Molecular Biology, School of Biology, Aristotle University of Thessaloniki, 54124 Thessaloniki, Greece. Phone: +306951626615. Email: tsarvale@bio.auth.gr


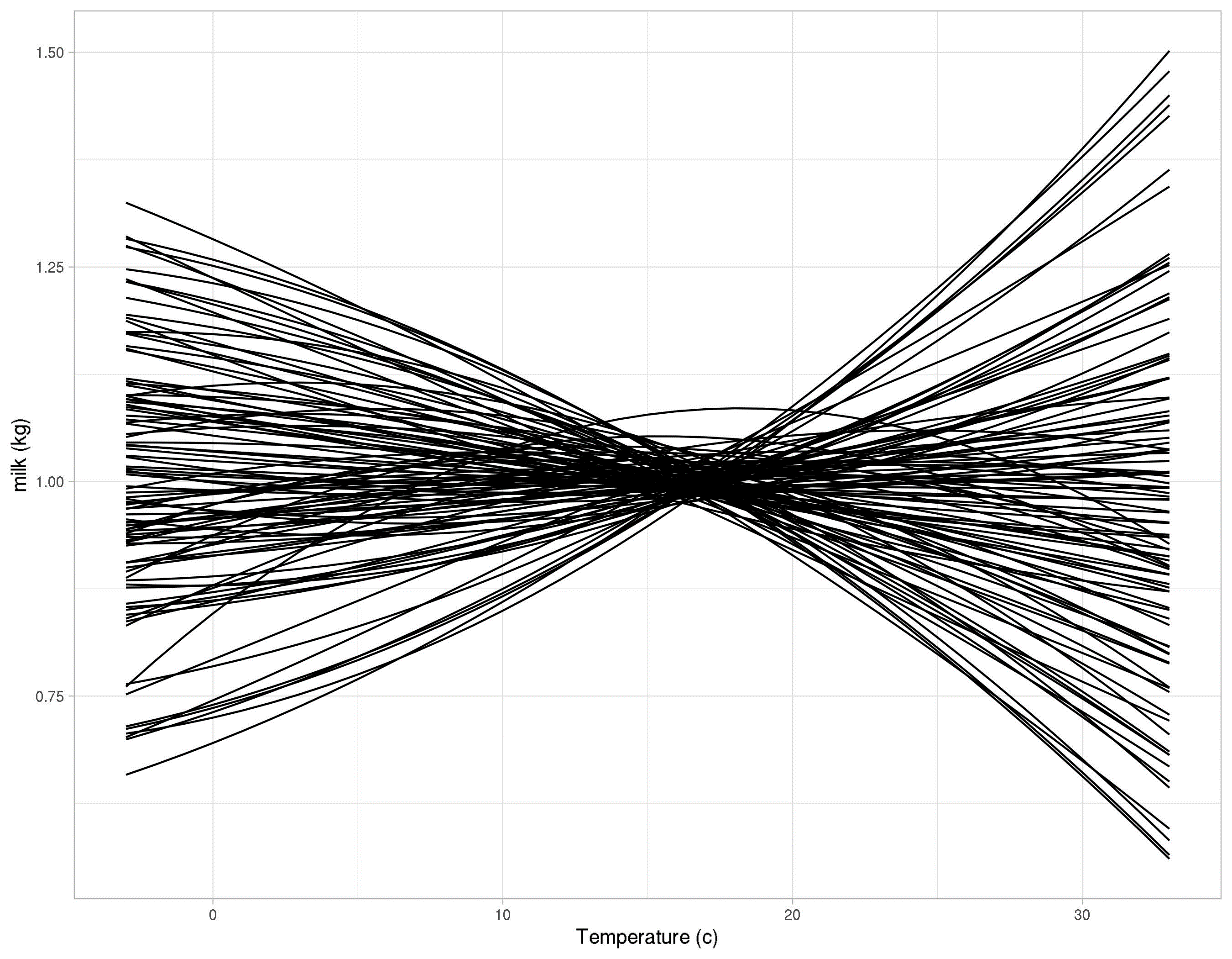
**Additional File 4: Figure S2**: Examples of individual animal resilience phenotypes manifested in reaction norm curves.

Each curve represents the individual animal change in daily milk yield (milk, kg) in response to average air temperature variation on the day of milk record (Temperature, ^o^C).
