# Supplementary material for: A comprehensive genome-wide scan detects genomic regions related to local adaptation and climate resilience in Mediterranean domestic sheep
Source: Genet Sel Evol. 2021 Dec 2;53:90. doi: 10.1186/s12711-021-00682-7 (PMC8641236; doi:10.1186/s12711-021-00682-7)
Supplement: Supplementary file 8 — Additional file 8: Figure S5. Protein interaction network. The present network represents the candidate identified genes for sheep resilience to temperature fluctuations under hot weather. [file 12711_2021_682_MOESM8_ESM.docx]

**A comprehensive genome-wide scan detects genomic regions related to local adaptation and climate resilience in Mediterranean domestic sheep**

Valentina Tsartsianidou^1*^, Enrique Sánchez-Molano^2^, Vanessa Varvara Kapsona^3^, Zoitsa Basdagianni^4^, Dimitrios Chatziplis^5^, Georgios Arsenos^6^, Alexandros Triantafyllidis^1^ & Georgios Banos^3,6^

^1^ Department of Genetics, Development & Molecular Biology, School of Biology, Aristotle University of Thessaloniki, 54124 Thessaloniki, Greece

^2^ Division of Genetics and Genomics, The Roslin Institute and Royal (Dick) School of Veterinary Studies, University of Edinburgh, Easter Bush, Midlothian EH25 9RG, UK

^3^ Department of Animal and Veterinary Sciences, Scotland’s Rural College, Roslin Institute Building, Easter Bush, Midlothian EH25 9RG, UK

^4^ Department of Animal Production, School of Agriculture, Aristotle University of Thessaloniki, 54124 Thessaloniki, Greece

^5^ Laboratory of Agrobiotechnology and Inspection of Agricultural Products, Department of Agriculture, International Hellenic University, Alexander Campus, 57400 Sindos, Greece

^6^ Laboratory of Animal Husbandry, School of Veterinary Medicine, Aristotle University of Thessaloniki, 54124 Thessaloniki, Greece

**Corresponding author:** Valentina Tsartsianidou. ^1^ Department of Genetics, Development & Molecular Biology, School of Biology, Aristotle University of Thessaloniki, 54124 Thessaloniki, Greece. Phone: +306951626615. Email: tsarvale@bio.auth.gr

**Additional File 8: Figure S5:** Protein interaction network.


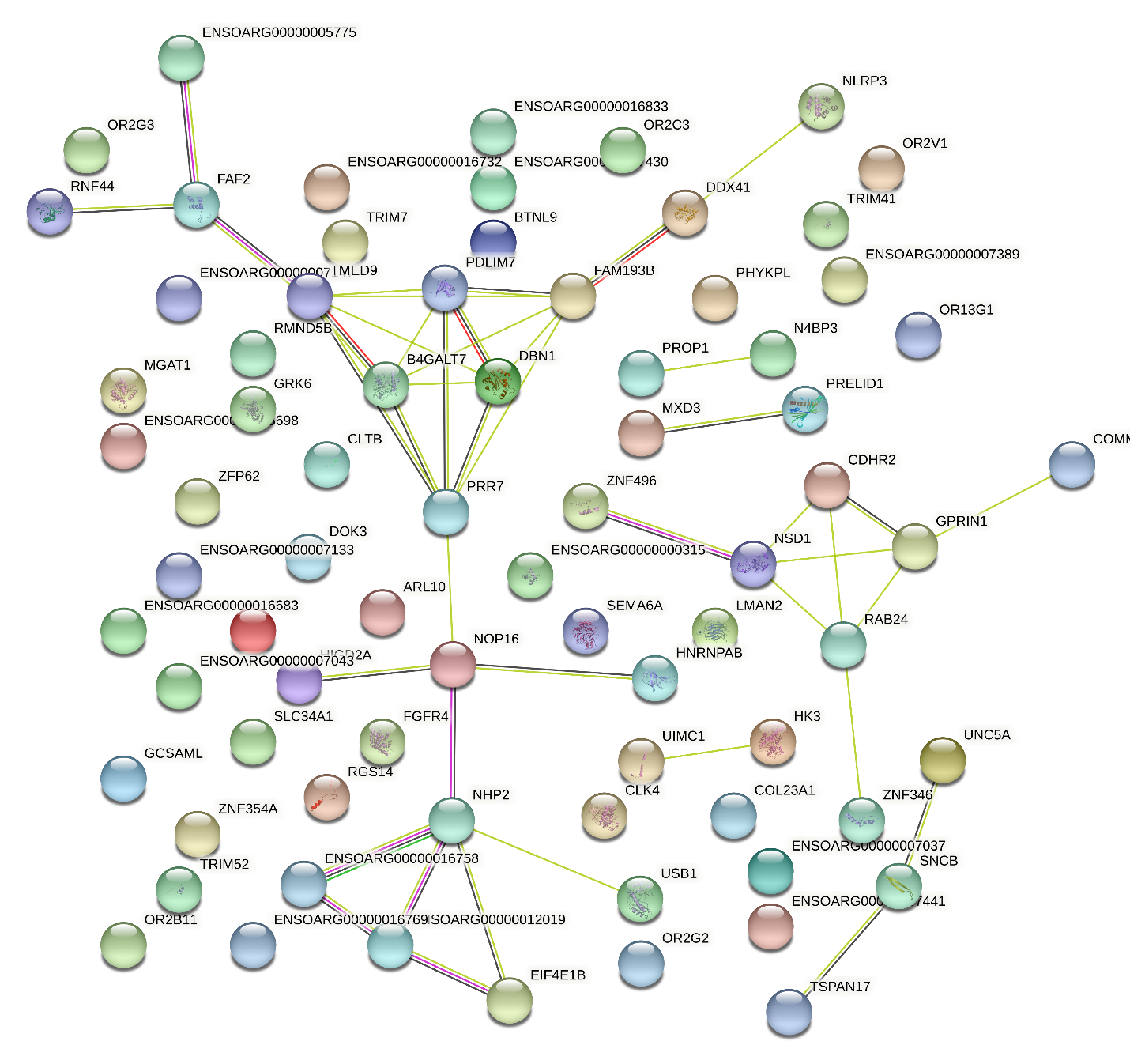


The present network represents the candidate identified genes for sheep resilience to temperature fluctuations under hot weather.
